# Adaptive Metabolism in Staphylococci: Survival and Persistence in Environmental and Clinical Settings

**DOI:** 10.1155/2018/1092632

**Published:** 2018-09-20

**Authors:** Laura A. Onyango, Mousa M. Alreshidi

**Affiliations:** ^1^Faculty of Natural & Applied Sciences, Trinity Western University, BC, Canada; ^2^Department of Biology, University of Hail, Saudi Arabia

## Abstract

Staphylococci are highly successful at colonizing a variety of dynamic environments, both nonpathogenic and those of clinical importance, and comprise the list of pathogens of global public health significance. Their remarkable survival and persistence can be attributed to a host of strategies, one of which is metabolic versatility—their ability to rapidly alter their metabolism in the presence of transient or long-term bacteriostatic and bactericidal conditions and facilitate cellular homeostasis. These attributes contribute to their widespread dissemination and challenging eradication particularly from clinical settings. The study of microbial behaviour at the metabolite level provides insight into mechanisms of survival and persistence under defined environmental and clinical conditions. This paper reviews the range of metabolic modulations that facilitate staphylococcal acclimatization and persistence in varying terrestrial and host conditions, and their public health ramifications in these settings.

## 1. Introduction

Staphylococci are important microorganisms influencing ecosystems, industry, animal, and human health. Their diverse lifestyles, whether as commensals or opportunistic pathogens, promote their prevalence in a wide range of environments, both nonpathogenic and pathogenic, where they can survive for short or extended periods, thus contributing to their spread and difficult eradication [1, 2]. Their involvement in a wide spectrum of community-acquired and nosocomial infections, some of which are highly recalcitrant to many clinical antibiotics, identifies them as a serious public health threat impacting morbidity and mortality rates and economies worldwide [3–8]. Their ability to successfully colonize niches and persist in spite of growth-limiting challenges, hostile host conditions, and even bactericidal measures has been attributed to many factors, including metabolic adaptation. Staphylococci can rapidly alter their physiology and cellular activities through metabolic modifications that enhance their fitness under these challenges, allowing their persistence and circulation between environments, and can also affect the nature of their pathogenesis [9–11]. Mechanisms by which staphylococci alter their metabolic profiles in adaptation and persistence have been investigated providing useful information on cellular function at an intricate level and may prove useful in finding novel targets that successfully inhibit microbial function and consequently make infection control more effective [12–15]. There are several studies exploring staphylococcal behaviour under various challenges. This paper will review the range of metabolic adaptations employed by staphylococci to overcome challenges and enhance persistence when exposed to anthropogenic, environmental, and host stressors.

## 2. Cell Envelope Modifications

The staphylococcal cell can alter several of its features to help adapt to environmental challenge and maintain homeostasis. The bacterial cell envelope (CE) (cell wall (CW) and cell membrane (CM)) is responsible for maintaining shape and turgor pressure, is involved in cell division, energy production, regulating permeability of substances in and out of the cell, and is involved in infection and pathogenicity [7]. This complex structure also plays an important role in adapting the cell under different conditions, transient or long-term.

### 2.1. Temperature-Induced Modifications

Adaptation to temperature changes is particularly crucial for staphylococci as they inhabit and circulate between many natural and man-made environments, several host species, varying anatomical sites, fomites, and food matrices, where they are susceptible to temperature fluctuations that can adversely impair the cell envelope and interfere with its intricate functions [16, 17]. The CW is the first point of contact between the bacterium and its external environment. Peptidoglycan is the major component of the CW and a crucial structure in stress survival and recalcitrance against antibiotics and host defences.

One of the most commonly observed stress responses in staphylococcal adaptation is CW thickening. Transmission electron microscopy (TEM) evaluations of* Staphylococcus aureus, Staphylococcus epidermidis,* and* Staphylococcus lugdunensis* clinical isolates exposed to 4°C for 8 weeks showed thickened CW structures associated with significant alterations in amino acid (AA) profiles in comparison to controls [12]. Modifications in AA content in relation to temperature adaptation were thought to be linked to an increase in CW-associated proteins, particularly cold-shock proteins which are essential in maintaining the integrity of this structure and functionality in cold temperatures [18–20]. The staphylococcal CM is also rich in fatty acids (FAs) and lipid content, essential to its adaptive functions in the presence of host defences, antimicrobial challenges, and acclimatization to environmental fluctuations [21]. FAs particularly help staphylococci prevent temperate-induced impairments in a process referred to as homeoviscous adaptation [22–24]. At optimal temperatures of 37°C, the CM of* S. aureus* is mainly composed of branched saturated FAs which determine membrane fluidity. As temperatures decrease, staphylococci modify the composition of their membrane to comprise mainly unsaturated FA, with monounsaturated FA being the predominant type. Additionally, synthesis of anteiso FA (C15:0) in preference of iso FA was observed. The incorporation of these lower melting point FAs into the membrane has been shown to significantly improve membrane fluidity in colder environments which maintains cellular function [25]. Mutants unable to synthesize these products due to the inactivation of the enzyme branched-chain *α*-ketoacid dehydrogenase (BKD) that catalyzes the production of FAs, not only showed impaired growth but also had less viscous membranes and were found to be highly susceptible in conditions of low temperatures [17]. Carotenoid pigments in* S. aureus* also play a crucial role in membrane stabilization under both cold and heat stress [7].

Staphylococci also display heightened thermotolerance which is crucial in instances when they are exposed to elevated ambient temperatures or subjected to sublethal temperatures employed in food pasteurization, for example, [26]. While the high temperatures utilized in food pasteurization effectively kill food-borne staphylococci, their preformed enterotoxins are resistant to heat-treatment and many other conditions and, when ingested in sufficient amounts, can cause acute gastrointestinal symptoms. Food-borne* S. aureus* is a known contaminant of a wide variety of foods when conditions allow its proliferation and subsequent toxin production [27]. A number of highly heat-stable* S. aureus* enterotoxins with super-antigenic activity have been identified in staphylococcal food-borne disease (SFD) which is a major public health concern worldwide. These enterotoxins are also resistant to the proteolytic enzymes and low pH environment of the digestive system [28].

Susceptibility to heat stress impairs bacterial growth, permeability, and can render cells vulnerable to other stress conditions [29–31].* S. aureus, S. epidermidis,* and* S. lugdunensis* all demonstrate high thermotolerance having been isolated from food treated at 80°C [32]. To maintain cellular homeostasis in extreme heat stress, staphylococci employ heat-shock proteins (Hsps) whose role in cellular metabolism is to protect against protein damage—misfolding and aggregation—and promote their refolding and proper assembly and movement across membranes [31]. A number of important Hsps identified in staphylococcal thermotolerance include Hsp 100, DnaK, and GroES/EL. Hsps normally expressed at temperatures of 30-37°C are abundantly expressed at elevated temperatures [31]. The increased expression of these Hsps is also observed in a range of other stress conditions that similarly cause protein unfolding [31, 33]. Hsps also play a role in staphylococcal pathogenesis. They aid bacteria in successful host colonization and infection, preparing the pathogen to resist the host's immune factors [31]. The synthesis of DnaK and GroEL was induced during infection, evidenced by antibodies detected in sera from patients with* S. aureus*-mediated endocarditis [34, 35].

### 2.2. Osmotic Pressure Induced Modifications

Staphylococci also display remarkable halotolerance in the presence of external osmotic pressure. Staphylococcal halotolerance makes these bacteria capable colonizers of environments with low water content and high salinity, which gives them a competitive advantage over many other microorganisms in these niches [36–38]. In human health and disease, halotolerance is particularly important for staphylococci as they comprise an abundant proportion of the skin microflora characterised by conditions of desiccation and high salt concentrations [39, 40]. Coagulase negative staphylococci (CNS) are more prevalent as skin commensals and* S. epidermidis* is best adapted to this environment, even contributing to the host's cutaneous defence repertoire against other invading pathogens [41, 42]. However, commensal staphylococci can also engage in an opportunistic lifestyle and cause a range of skin infections and other difficult-to-treat infections [27].

To observe the adaptive mechanisms involved in osmotic-stress tolerance, staphylococci have been exposed to NaCl-rich media. The initial shock of increased external osmotic pressure caused* S. aureus* cells to lose water resulting in lowered turgor pressure and cell shrinkage [43]. NaCl-sensitive cells unable to compensate for these adversities progressively exhibited retarded growth, impaired daughter-cell separation, and atypical CW associated with the inhibitory effects of osmotic stress. In contrast, salt-tolerant cells (isolated from fermented seafood) exhibited significantly larger cell sizes, tetrad/cubical cell, and significantly thicker CW. These features are thought to help alleviate water efflux and other inhibitory effects of osmotic stress by reducing surface area-to-volume ratio [29–33]. Using high-performance liquid chromatography (HPLC), CW profiles of NaCl-stressed* S. aureus* cells showed reduced amounts of penta-glycine residues. NaCl stress disrupts the process of glycine addition during peptidoglycan assembly resulting in shorter interpeptide bridges and fewer cross-linkages between the muropeptide layers and a reduction in the synthesis of CW-associated proteins [43]. The combined effect is a loosely linked peptidoglycan layer that is less susceptible to the hydrolases that facilitate cleavage and turnover, resulting in thicker CW [44]. Solid-state nuclear magnetic resonance (NMR) spectroscopy examinations revealed that NaCl stress did not affect already formed peptidoglycan strands, only the newly synthesized peptidoglycan [45]. Alterations in staphylococcal membrane phospholipid content have also been observed in response to changes in salinity [7].* S. epidermidis* isolates incubated in media with NaCl concentrations <15% showed no significant changes in their FA composition. However, the proportion of anteiso-FA (C15:0) was greatly increased when the concentration was increased to 25% NaCl [46]. Staphylococcal osmotic-stress adaptability also confers a ‘cross-protection' against the effect of antibiotic compounds. Food-borne staphylococci isolated from fermented foods with concentrations of 11% NaCl were found resistant to a bacteriocin-like compound [37].* S. aureus* isolated from a kitchen and exposed to 4% NaCl concentrations became less susceptible to conventional antibiotics, with a reported fourfold decreased sensitivity, which was maintained even after stress removal [29]. A similar finding was seen in clinical samples of* S. lugdunensis* which became highly resistant to gentamicin when grown in the presence of 5% salt concentrations [14]. Adaptations to one stress that confers a cross-protective effect against antibiotic stress complicate the management of antibiotic resistance (ABR) in these pathogens [29]. With regard to staphylococcal virulence factors, however, exposure to high salt concentrations appeared to repress their expression rendering these pathogens less virulent [47].

### 2.3. Antibiotic-Induced Modifications

Although the antibiotic era is only seven decades old, antibiotics have long existed in the natural environment and bacteria have developed mechanisms to circumvent their range of effects. The misuse and overuse of antibiotics in clinical, agricultural, and industrial settings have exacerbated the prevalence of resistant bacteria, undermining their clinical efficacy worldwide.

The staphylococcal CW is a major target of antibiotic therapy. CW-active antibiotics act by interfering with one or more of the steps involved in CW synthesis and assembly. In staphylococci, vancomycin, for example, inhibits the second stage of CW synthesis, blocking the transglycosylation step, which subsequently affects transpeptidation [48, 49]. However, many staphylococci are equipped with strategies that circumvent these effects.* S. aureus* cells exposed to vancomycin exhibited accelerated uptake of the N-acetylglucosamine (NAG) subunit into the cell, halted or delayed CW turnover, increased proportions of glutamine-nonamidated peptidoglycan subunits, and decreased peptidoglycan cross-linkages. These modifications lead to thickened CWs that confer protection against the antibiotic. The periphery of the thickened wall acts as a false target that sequesters and confines the active antibiotic, distancing it from reaching its actual target (the site of CW biosynthesis close to the plasma membrane). The poor cross-linkages also significantly increase the proportion of free carboxyl termini that can bind the antibiotic also preventing it from the target. Once the antibiotic threat abates, studies showed that the cells recover, and through autolytic processes they disintegrated the thickened CW giving way to conventional formation of a new CW [50–52]. The bacteria also slow the rate of growth and inhibit daughter-cell separation leading to much larger cell sizes than normal [51]. Hsps are also thought to play an important role in antibiotic tolerance as their amounts are elevated during exposure of the bacterium to CW-active antibiotics in order to counteract the action of the antibiotic. Mutants incapable of producing these proteins are highly susceptible to the effect of the antibiotic, thereby greatly reducing their rate of survival [22, 23]. Proteomic studies showed that although synthesis of peptidoglycan and overall protein production were generally inhibited by the action of the antibiotic in* S. aureus,* production of a specific set of nine proteins was sustained and significantly increased in these cells. This increase was hypothesized to relate to the overproduction of polypeptides involved in CW biosynthesis. These proteins shared homology with others that function as enzymes, signal transducers, and Hsps. This proteomic signature in* S. aureus* in response to antibiotic stress was presumed to counteract the lethal effects of the antibiotics and maintain envelope integrity [51, 53].

When vancomycin is no longer effective in chemotherapy, particularly for MRSA infections, a membrane-active antibiotic like daptomycin is employed as the next course of action. Daptomycin binds to and alters the shape of the membrane leading to the formation of pores that allow ions to leak in and out of the cell. The result is a loss of membrane potential and the inhibition of RNA, DNA, and protein synthesis, eventuating in cell death [54, 55]. The transition from a susceptible to nonsusceptible staphylococcal phenotype in daptomycin tolerance was associated with growth retardation during the postexponential phase, decreased TCA cycle activity with an increased redirection of carbon flow to pathways associated with biosynthesis of CW components (wall teichoic acids and peptidoglycan (thicker peptidoglycan)), and increased synthesis of nucleotides (pyrimidines and purines) [54].

Studies exploring the global impact of antibiotics on the metabolome of* S. aureus* exposed this species to five antibiotics (ciprofloxacin, erythromycin, fosfomycin, vancomycin, and ampicillin) over a 2 hr growth period and the metabolite shifts were analysed. Using a combination of H-NMR spectroscopy and GC/LC-MS techniques, a total of 214 metabolites (38 extracellular and 176 intracellular) were detectable. Although the antibiotics exert action in different ways, each antibiotic elicited a metabolome-wide stress response in* S. aureus* with an overlap of effect in other pathways. The peptidoglycan pathway and purine and pyrimidine metabolism showed the most alterations. Intermediates of the tricarboxylic cycle (TCC) were also affected by every antibiotic tested revealing the importance of this cycle in* S. aureus* stress response [56].

### 2.4. Other CE Modulations

Cardiolipin (CL), a membrane phospholipid, has been associated with bacterial adaptability to several stressors [57]. An increase in CL content was observed when* S. aureus* was exposed to high salt concentrations [58]. Actively growing* S. aureus* cells did not require CL to grow when exposed to high salt concentrations but CL was essential for the long-term fitness of* S. aureus* in high salinity [59]. During active growth,* S. aureus* cells have phosphatidylglycerol (PG) as their main membrane phospholipid. As the cells enter stationary phase, an increase in CL was observed while the content of membrane PG decreases. PG is a precursor of CL, which during stationary phase is converted to CL resulting in the observed phospholipid alterations [57, 60]. Other minor lipids such as phosphatidylethanolamine and phosphatidyl-glucose are also increased in stationary phase, but lysyl-phosphatidylglycerol (L-PG) content remains unchanged [7]. CL was also found to be essential in antibiotic-stress response.* S. aureus* cells treated with CW-active antibiotics dissolved in 0.85% NaCl solution, showed increased content of CL in their phospholipid extracts [61]. Greater CL increase was observed in cultures treated with nonbacteriolytic antibiotics (e.g., penicillin) in comparison to those exposed to bacteriolytic antibiotics (e.g., bacitracin) (16-27% versus 55.2-71%). In this study, CL was thought to prevent* S. aureus* cell lysis by inhibiting the activity of the enzyme autolysin as well as maintaining the rigidity of the CW [61]. The use of bacteriostatic agents in bacterial control such as those employed in food preservation has been shown to impede on the efficacy of some antibiotics, a result suggesting that these methods may contribute to heightened resistance and aid dissemination of pathogenic strains [29].

The role of membrane lipids in long-term survivability of CNS has also been investigated. CNS are adept at colonizing and persisting on medical devices and have been associated with numerous recalcitrant nosocomial infections [99]. Using thin-layer chromatography (TLC) and MS, the exponential phase lipid extracts of both* S. epidermidis* and* S. haemolyticus* were found to be relatively similar, both abundant in diacylglycerol (DAG), PG, L-PG, and diglucosyl-diacylglycerol (DGDG). Alanyl-PG content was relatively low in both bacteria, while N-succinyl-lysyl-PG was detected only in* S. haemolyticus* and lysyl-DAG only in* S. epidermidis*. The transition into stationary phase resulted in significantly altered profiles for both bacteria. In addition to arresting their growth, the synthesis of aminoacylated phospholipids ceased in both bacteria, with both accumulating free FA from continued FA synthesis during this phase. In addition, CL was abundantly present in* S. epidermidis,* which in other starved model organisms has been shown to be essential to their survival during nutrient depletion.* S. haemolyticus* simplified its profile modulations accumulating the membrane lipids DGDG and PG, with no noticeable CL production [62]. Surviving nutrient deprivation is important in the persistence of these clinical isolates in instances of transfer from host sites and circulation between other environs.

## 3. Cytoplasmic Modulations

While the cell envelope provides significant protection and adaptability for the bacterium in the presence of external adversities, cytoplasmic-level modulations are also possible in the event of challenge and envelope compromise.

### 3.1. Temperature-Induced Changes

*S. aureus *cells exposed to cold temperature showed a significant upregulation of ten ribosomal proteins and a reduction in cytoplasmic AA concentrations in these samples. Ribosomal proteins act as temperature sensors and the upregulation in their synthesis in this study reflects their role in staphylococcal acclimatization to prolonged instances in cold temperature [13].

### 3.2. Osmoprotection

Halotolerant staphylococci can also adapt their cytoplasmic content to maintain osmotic pressure in the presence of a broad range of salt concentrations. As previously mentioned, high salt concentrations in the external environment can severely dehydrate the cell and interfere with turgor pressure but can also affect DNA replication and the structure of many cytoplasmic proteins and their associated functions. To circumvent these adversities, bacteria can employ halophilic proteins that maintain soluble and active conformations in these conditions via protein haloadaptation [63]. NaCl-tolerant staphylococci possess a high number of genes associated with AA transport and metabolism in conditions of high osmotic strength. This assists in heightening osmoadaptive activity, which enables staphylococci to efficiently transport or synthesize osmotically active solutes within their cytoplasm to help survive dehydration without interfering with normal cytoplasmic activities under these conditions [38, 64, 65]. Proline and glycine betaine are examples of effective osmoprotectants that accumulate within staphylococcal cells in environments of high osmotic stress [15, 65]. When exponentially growing* S. aureus* cells are introduced into high salt concentrations, a growth lag of 15 min was observed as well as cell shrinkage. Within half an hour of incubation in high salt medium, intracellular content of free AA was noted, with proline being the most prominent [66]. Glutamic acid, asparagine, and glutamine levels also increased slightly but gradually receded to normal levels. The active uptake of proline concurrently increased intracellular water content. The efficacy of glycine betaine in restoring normal cellular morphology and growth was demonstrated in a NaCl-sensitive mutant [65, 67, 68]. Other compatible solutes which are also accumulated in high NaCl conditions include glutamine, alanine, choline, proline betaine, taurine, and glutamic acid [67, 69]. While* S. aureus *synthesizes molecules such as glutamic acid, other solutes are imported from the external environment via transport systems. Mutations that inhibit functionality of these transport systems in* S. aureus* reduced its survivability in infection models at locations of high salt concentrations [69].

### 3.3. Nutritional Adaptations

Staphylococci also encounter numerous nutrient-limiting environments as they circulate between terrestrial habitats, clinical settings, and host species. Nutrient deprivation can severely affect critical cellular functions and impede viability. However, staphylococci successfully overcome these challenges and can persist for extended periods in these environs. In clinical settings, staphylococci can remain viable on hands, in the air, in sterile solutions, on surfaces, on medical devices, and many more for extended durations allowing for their dissemination and transfer, which has greatly contributed to their prevalence and persistence in nosocomial infections and grossly undermines infection control strategies and affects patient outcomes. As pathogens of significant public health concern, understanding the starvation-survival strategies that staphylococci employ in their persistence can aid in better clinical practices and the development of novel therapeutic strategies in combating staphylococcal infections.

The survival potential of staphylococci under several nutrient-limiting conditions has been investigated. Glucose is a major source of carbon for staphylococcal growth.* S. aureus* grown in glucose-limiting media lost majority of the population leaving a very small remnant of cells with heightened long-term survival potential. These cells were smaller and exhibited increased resistance to certain stress conditions. The transition into glucose-starvation survival was accompanied by protein synthesis which was maintained into long-term starvation survival. The role of these proteins included nutrient scavenging and recovery from starvation [70]. Another study investigating glucose-limitations on* S. aureus* growth observed a significant reduction in protein synthesis during the initial stages of starvation. Proteolysis of enzymes involved in several metabolic processes and pathways were observed as cells entered stationary phase as well as the uptake of the AA alanine and glycine. Proteins no longer required in nongrowing cells were degraded to supply and replenish nutrients required by starvation survivors [71].* S. aureus* can also make use of metabolites such as acetate, pyruvate, and succinate which are secreted during exponential growth as options following glucose depletion from the medium. Alterations in the intracellular concentrations of free AA were also observed in glucose starvation. Growing* S. aureus* cells that were supplemented with 15 AA and grown into starvation stage accumulated AA rather than undergo* de novo* synthesis. The uptake of lysine, histidine, cysteine, and aspartate, for example, increased substantially during starvation [72]. An abundance of AA is essential for protein biosynthesis for long-term survivability [62]. In general,* S. aureus* copes with nutrient-limiting situations by adjusting its metabolism to levels needed by the cell to survive and persist extracellularly.

Once intracellular, staphylococci also encounter host-mediated nutritional restrictions that limit the supply of critical substances from infection sites via a mechanism referred to as nutritional immunity [73]. Important nutrients include metals such as iron, manganese, and zinc, which are essential in a range of metabolic processes that facilitate staphylococcal growth and proliferation [74]. Neutrophils function to restrict staphylococcal access to these metals during infection via various mechanisms [75].* S. aureus* employs a multifaceted approach to maintain metal homeostasis intracellularly. Bacterial siderophores such as staphyloferrin A and B, for example, compete with host systems to bind extracellular iron. Siderophore-mediated iron acquisition is crucial for* S. aureus in vivo *but the same mechanism promotes its growth* in vitro *[76].* S. aureus* can also acquire iron from host heme, which is a much richer source of iron. Using pore-forming toxins,* S. aureus* lyses host erythrocytes releasing heme which is then bound and moved into the cytoplasm by heme-acquisition mechanisms, where it is degraded to release free iron [77].* S. aureus'* ability to acquire iron via either siderophores or heme-acquisition systems contributes to its virulence* in vivo.* Heme-binding proteins contribute to staphylococcal adhesion to host tissues and its resistance to innate immune processes [78].

Intracellular staphylococci also have to contend with the host's oxidative immune response which can affect cellular AA, proteins, and DNA with bactericidal consequences. Staphylococci have developed a repertoire of mechanisms that can detect, protect against, detoxify, and repair the effects of host oxidative stress [74]. Examples include the membrane-bound carotenoid pigment staphyloxanthin that gives* S. aureus* colonies its golden-yellow colour and is a potent antioxidant that detoxifies reactive oxygen species (ROS) and aids in intracellular virulence and persistence. Manganese is an important metal ion that is also involved in detoxification of ROS and also promotes intracellular virulence. Host phagocytic cells hinder the availability of manganese from engulfed bacteria by transporting it out of the phagosome or chelating it. To circumvent this action and fulfill its manganese requirement,* S. aureus* utilizes manganese transporters. To repair DNA damage owing to oxidative stress, the global SOS response system is activated and has proven highly effective in repairing damage caused by this and many other challenges [74]. This system has also been associated with staphylococcal virulence and resistance, and even the phenotypic switching of staphylococci during the DNA repair process [79].

## 4. Population Adaptations

Under controlled experimental conditions where bacteria are cultured in optimal, nonstress parameters, population homogeneity is the main mode of existence [29]. However, in the free-living natural environment where perturbations are the norm, bacteria can exist as a diverse, heterogeneous population [80]. Examples of population heterogeneity in staphylococci include the formation of phenotypic variants such as persister cells and small colony variants (SCV). Both phenotypes share many similarities including their atypical metabolism, heightened tolerance to a range of terrestrial and host stressors, and involvement in many recalcitrant infections [19, 81].

### 4.1. Persister Cells

Staphylococcal persister cells were first described in the 1940s in association with penicillin use, when a population of actively growing* S. aureus* were lysed by the antibiotic, but a small remnant of cells persisted [82]. Since that time, persister cells have been documented for many other bacteria exposed to different classes of antibiotics [81, 83] and a range of other environmental conditions and hazards [83–85]. By definition, persister cells are a subpopulation of a genetically homogenous culture; they display phenotypic variations which enable them to tolerate and endure selective pressures that other members of the population are susceptible to [86]. In natural environments, these specialised survivor cells are thought to be preexisting through stochastic formation, as an evolutionary mechanism to maximize the survival fitness of the population under a wide variety of challenges [87]. Persister formation has also been attributed to a responsive mechanism where the bacteria respond to environmental cues by quantitatively and qualitatively modulating the rate at which their members undergo phenotypic conversion [84]. The mechanisms that facilitate phenotypic heterogeneity are epigenetic in nature, not involving changes in the underlying genome, thus allowing phenotypic reversibility in stress-free subculture. Understanding the underlying processes that enable phenotypic persistence is important to help improve treatment strategies, curb resistance development, and improve patient prognosis.

Staphylococcal persister cells display several metabolic characteristics that promote their survival and persistence in adverse environments. Classical descriptions of persister cells characterised them as dormant—metabolically inactive—but evidence shows that these cells remain active, albeit with minimal metabolic capacity that can maintain viability even for prolonged durations. In this way, persister cells possess similarities with stationary-phase cells that also undergo arrested growth and replication, and can sustain this state for a long-term. Transition into a persister phenotype has been characterised by fluctuations in specialised persister proteins [88], a reduction of intracellular adenosine triphosphate (ATP), and the expression of stationary-state markers [89]. When exposed to antibiotics that inhibit peptidoglycan synthesis,* S. aureus* persisters decreased membrane fluidity by increasing their membrane saturated-FA content while concurrently reducing branched-FA content. Persister cells maintained oxygen consumption in the presence of CW-active antibiotics although the rate was significantly reduced. These cultures also showed evidence of enzymatic activation related to lipid, protein, and saccharide metabolism [90]. These changes impact cellular processes including growth, cell division, and physiology [88]. Maintaining a level of metabolic activity during persister quiescence is pertinent to cellular integrity long-term and subsequent resuscitation and return to normal function when challenges abate. Because persister phenotypic tolerance is unlike resistance which is genetically determined, persister characteristics were considered to be nonheritable by future progeny [91]. However, some studies have observed transmission of antibiotic tolerance to daughter cells maintained for several generations. This transgenerational plasticity is attributed to mechanisms of epigenetic inheritance [87, 90].

The presence of persister phenotypes in clinical settings presents problematic implications. Their general downregulation of cellular activities greatly hampers the action of many chemotherapeutic agents that depend on active targets. Their altered metabolism also adapts them to the host's intracellular milieu and impedes their clearance particularly when they exist in biofilm structures. In fact, the heightened antibiotic tolerance associated with biofilm structures has been directly attributed to the presence of persister cells housed within. These cells can withstand prolonged and elevated concentrations of antibiotics that readily penetrate the biofilm core [86]. The transient nature of persisters allows them to regain a fully functional metabolism where they can replenish the population and reinitiate infections creating a cycle which can prove difficult to resolve [83]. These factors not only limit treatment options but also prolong treatment regimens which can subsequently promote the development of antibiotic resistant strains and further lead to treatment failure. Current research efforts are exploring the development of measures that address the persister lifestyle which includes preventing the transition of cells into quiescence, targeting the processes that facilitate resuscitation into a fully metabolic state, and the use of substances that awaken cellular targets for antimicrobial action [92].

### 4.2. Small Colony Variants (SCVs)

Staphylococcal SCVs were first observed in the early 20th century and have gained reputation owing to their involvement in numerous recurrent nosocomial infections. They are a naturally occurring, slow-growing bacterial subpopulation which have been isolated under various conditions, and exhibit atypical morphological, ultrastructural, and biochemical properties in comparison to their corresponding wildtype (WT) that render them well-suited to long-term survival both terrestrially and intracellularly [93].

Metabolically, SCVs have been described as auxotrophs of haemin, menadione, and thymidine. Deficiencies in the production of these substances alter the electron transport chain (ETC) coupled with poor utilization of carbon sources and affect ATP production, resulting in slower growth rates, altered cell division, CW biosynthesis, AA transport and protein synthesis, decreased membrane potential, cationic and peptide transport, and carotenoid synthesis [94–96]. SCVs also display a significant reduction in biosynthesis of proteins related to the tricarboxylic acid (TCA) cycle, purine/pyrimidine, arginine and proline synthesis, and folate metabolism [97] and a downregulation of the activity of their citric acid cycle [98]. The alterations in morphology and biochemistry greatly impede accurate detection by conventional identification techniques which depend on these features, resulting in inaccurate diagnosis and treatment [93]. Similar to persister cells, SCVs are highly recalcitrant to many clinically important antibiotics not only because their altered metabolism reduces the efficacy of agents that depend on metabolically active targets, but also because SCV can also tolerate significantly elevated levels of many other antibiotics that do not depend on metabolic activity. This severely limits therapeutic options and often results in a poor clinical outcome for patients [99].

The SCV phenotype is armed with a host of features that suitably adapt it to intracellular living. Their decreased production in antigenic fragments masks their detection by host immune elements inhibiting rapid clearance and allowing their circulation from site to site. Their downregulation of factors associated with WT virulence such as decreased toxin production has less cytotoxic damage on host cells and ensures a prolonged niche for their persistence [100, 101]. Their increased expression of fibronectin-binding proteins, usually associated with cellular invasion, aids them adhere more to host cells and facilitate rapid internalisation. Once internalised, SCVs withstand the milieu by upregulating protective mechanisms highly effective in maintaining viability [100, 102]. An upregulated arginine-deiminase pathway, for example, compensates for defects in ATP production and counteracts the intracellular acidic environment [94].

Like persister cells, staphylococcal SCVs are also associated with biofilm structures. Studies showed that SCV biofilms were generated much faster and were significantly thicker than their corresponding WT owing to upregulation of capsular polysaccharide synthesis [103]. This structure is a crucial virulence factor particularly for CNS as it aids in their adherence and surface colonization in device-related infections [104]. The combination of a SCV phenotype and hyperbiofilm formation results in a bacterial mass that is highly adept at adhering to and sustaining relapsing implant-related infections [105, 106]. These are often difficult to resolve with chemotherapy and often require complete removal of the medical device to be resolved. Because SCVs are phenotypically determined, their characteristics are transient and, thus, they are able to revert to their WT phenotype when conditions permit. The switch between phenotypes (WT⬌SCV) contributes to resurgence of infections, even years after the initial incident. Overall, the SCV phenotype is a cost-effective strategy that enhances bacterial persistence in a range of conditions, and for pathogens like the staphylococci, having this phenotype as part of their lifestyle is highly advantageous [107].

## 5. Conclusion

Staphylococci remain ubiquitous in the environment in spite of many conditions not propitious for their growth and proliferation. Their involvement in a wide spectrum of infections, some of which are difficult-to-treat, has earned them global recognition as one of the most notable pathogens of public health significance. Their remarkable survivability and persistence amidst the physicochemical pressures present both intracellularly and externally are made possible in part by their metabolic versatility—their ability to modulate their metabolic processes and composition to overcome challenges. This attribute has contributed to their vast dissemination and resistance development, and highlights their problematic eradication in clinical settings.

Adaptive metabolism in staphylococci appeared to be well-developed and a cost-effective strategy, attuned to ensure survival and competitiveness in a range of situations, both externally and intracellularly. While some responses appeared to be similar across the staphylococci, others were unique to the particular strain. Commonly detected metabolite shifts were seen in AA, FA, and phospholipid content. In most cases reviewed, the synthesis of these adaptive metabolites increased under stress and functioned to help maintain homeostasis and enhance persistence in a challenge. Some of these changes were manifested as physiological and ultrastructural modifications of target sites, for example, CW thickening, which appeared to be a more universal adaptive response to several challenges. It was also noted in some instances that adaptation to one stress conferred a cross-protection to another condition demonstrating cost-effectiveness. Other more advanced adaptations such as population heterogeneity involved broader transformations including the transition from normal growth to metabolic quiescence—modulation of processes and metabolites, in favour of only what was pertinent to staphylococcal survival, persistence, and subsequent resuscitation in that environment.

The ability of staphylococci to exist as subpopulations of phenotypic variants with heightened physiological tolerance and downregulated metabolic processes is also cost-effective, maximizing the fitness of the population and ensuring long-term survivability under a range of both bacteriostatic and bactericidal pressures. This heterogeneity promotes their colonization of various niches, including those of the human host, and even alters the pathogenic profile to reflect their evolution, such as what is seen with SCV infections. Both persister and SCV phenotypes have proven problematic to eradicate and, when associated with pathogens like the staphylococci, these phenotypes enhance and complicate their public health impacts, particularly in the chronicity of infection and treatment failure. Variations in staphylococcal antibiotic susceptibility as a result of adaptive tolerance by both these phenotypes also prolong their presence in the environment where they can also acquire resistance genes and act as a reservoir for the dissemination of these genes, thereby promoting the development of antibiotic resistance in new niches even those devoid of antibiotics.

The study of adaptive metabolism in staphylococci is important in understanding staphylococcal behaviour and consequently developing approaches to target the strategies that render them problematic in various settings. Future investigations in this field would include approaches that perturb staphylococcal transitions. Inhibiting the specific adaptive metabolites or the pathways that facilitate these metabolic shifts and phenotypic transformations provides a viable alternative that circumvents use of chemotherapeutics that promote the development of classical resistance mechanisms. Since alterations in staphylococcal metabolism affect accurate diagnosis particularly where conventional tools that depend on a specified biochemistry are utilized as the first line of investigation and perhaps without additional confirmation, there is a need to develop novel tools that can rapidly detect altered metabolic states or atypical phenotypes to enable accurate and timely diagnosis and appropriate clinical intervention. Through this review, it was noted that the majority of staphylococcal metabolic studies were understandably focused on* S. aureus* owing to its involvement in numerous difficult-to-treat infections. However, there is a need to extend these investigations to encompass other members of the staphylococci that have also gained clinical prominence. CNS are increasingly involved in nosocomial infections particularly those associated with indwelling medical devices and biofilm. This approach would provide a more comprehensive understanding of combating staphylococcal infections.
